# Perceptions about sexual abstinence and knowledge of HIV/AIDS prevention among in-school adolescents in a western Nigerian city

**DOI:** 10.1186/1471-2458-11-304

**Published:** 2011-05-12

**Authors:** Oladimeji Oladepo, Mojisola M Fayemi

**Affiliations:** 1 Department of Health Promotion & Education, Faculty of Public Health, College of Medicine, University of Ibadan, Nigeria; 2 Association for Reproductive & Family Health, Quarters 815 A Army Officers Mess Road, Ikolaba, Ibadan, Nigeria

**Keywords:** Adolescent, Sexual Abstinence, Premarital Sex, HIV/AIDS

## Abstract

**Background:**

Young people are becoming increasingly exposed to the risk of HIV infection. According to the 2008 HIV/Syphilis sentinel survey in Nigeria, 3.3% of young people aged 15-19 years are infected. Primary prevention especially abstinence, remains one of the most realistic interventions for reducing further spread of the virus. However, the adoption of sexual abstinence as a prevention strategy among adolescents remains low and factors influencing its practice among urban young people in Nigeria are relatively unknown. The aim of the study was to document the sexual abstinence behaviour of in-school adolescents, the factors influencing or obstructing abstinence, and knowledge of HIV and AIDS in Ibadan, South-West Nigeria.

**Methods:**

The study was a descriptive cross-sectional survey of students in Ibadan South-West Local Government Area. A total of 420 respondents (52% males and 48% females), selected through a multistage sampling technique, completed a semi-structured questionnaire. This was supplemented with eight focus group discussions (FGDs) which had an average of 9 respondents within the 10 and 19 years age group. The data from the FGDs were transcribed and summarized manually while the quantitative data was analyzed using the Statistical Package for the Social Sciences to generate frequencies, cross tabulations of variables and logistic regression analysis.

**Results:**

Twelve percent of the entire sample had ever had sex. Overall, knowledge of HIV transmission and prevention was high and most respondents favoured the promotion of abstinence as an HIV prevention strategy. A smaller proportion of male respondents (79%) abstained compared with the females (98%). Major predictors of sexual abstinence were being a female, not having a boyfriend or girl friend, not using alcohol and having a positive attitude towards abstinence (P < 0.05).

Sexual abstinence was also significantly associated with perceived self efficacy to refuse sex and negative perception of peers who engage in sexual behaviours (P < 0.05). Majority of the FGD discussants suggested the involvement of parents, media, schools, faith-based institutions and non governmental organizations in promoting the adoption of abstinence.

**Conclusions:**

The sexual abstinence behaviour of young persons is influenced by multiple factors and should be considered in determining the effectiveness of interventions targeting this behaviour. Coherent sexuality education interventions to promote the adoption of abstinence among young people are urgently needed.

## Background

Human Immunodeficiency Virus has emerged as a major health and development concern worldwide. Globally, 33.2 million people were estimated to be living with HIV and 2.5 million were newly infected with HIV in 2007 [1]. As the HIV epidemic spreads, younger age groups are becoming exposed to the risk of infection [2]. Ten million youth (ages 15-24) worldwide are living with HIV and every day, an estimated 6,000 youth are infected with the virus [3]. The 2008 HIV/Syphilis Sentinel Survey in Nigeria revealed that 3.3% of young people aged 15-19 are infected with the HIV virus [4]. Sexual intercourse is the most predominant mode of transmission of HIV in sub-Saharan Africa, accounting for approximately 90% of all infections [5].

Young people are particularly vulnerable to HIV infection because of the physical, psychological, social and economic attributes of adolescence [6] and are also at risk due to the high levels of risky sexual behaviours and the attitudes, expectations and limitations of the societies in which they grow up [7]. Studies have shown that, adolescents who begin sexual activity early are likely to have sex with more partners, and with partners who have been at risk of HIV exposure [8]. Thus, a major goal of HIV prevention programs is to delay sexual debut.

Individual behaviour change, especially sexual behaviour change, appears to be the most effective means to prevent further spread of HIV in Africa [9]. Uganda has been able to halt and reverse the HIV pandemic through individual behaviour change - *abstinence, being faithful and condom use *[10]. Though there are diverse opinions on the role and effectiveness of abstinence only programme in reducing Uganda's HIV prevalence, [10-12] indication that it may have played an important role has renewed an interest in promoting this method of protection against unplanned pregnancy, HIV and other sexually transmitted infections.

Although abstinence and condom use have been recommended as measures for controlling the spread of HIV/AIDS among adolescents [13] their use as prevention strategies in Nigeria remain low [14]. The National HIV/AIDS and Reproductive Health Survey showed that only 47% of females and 27% of males aged 15-24 reported abstinence [14]. Traditional norms in most Nigerian culture demand sexual abstinence before marital unions. However, due to the increase in the age at marriage, such norms have almost disappeared in all ethnic groups [15,16]. According to the 2008 Nigeria Demographic Health Survey, only 12% of women aged 15-19 had been married by age 15 and 39% of women 20-24 were married by age 18.

Studies conducted in some countries revealed that factors which militate against adolescents abstaining include the influence of the media which brazenly endorses sex, low self esteem and personal values, desire to conform to perceived peer's premarital sexual activity and difference between the age of puberty and age of marriage [17-20]. Other factors which influence the adoption of abstinence by young people include unequal gender norms [21], parental influence [22], poverty [23], alcohol use, perceived self efficacy to refuse sex [24], attitude towards premarital sex [25] and religiosity [26].

The National Policy on health & development of adolescents & young people in Nigeria promotes abstinence only programmes as a key intervention for young people [27]. However, evidences from systematic reviews conducted in developed and developing countries suggest that abstinence only programmes to prevent HIV infection are ineffective and do not effectively encourage abstinent behaviour [28,29]. On the other hand, it is difficult to reach any definitive conclusion about their impact because few studies have been conducted to assess its effectiveness. This does not mean that these programs are not effective; but too little evidence exists to reach any strong conclusion about their effectiveness [30].

In Nigeria, condom promotion has continued to face religious, logistic, social and economic obstacles [[31,32], and [33]]. This underscores the need to increase support for comprehensive programmes which emphasize abstinence at the same level with condom promotion and address not only knowledge or attitude change, but the social context in which young people live and make decisions [34].

In order to develop effective comprehensive sexuality interventions for young people in Nigeria, there is need to identify factors and the social context which influences their adoption of abstinence. Thus, further research is required to provide insightful information to policy makers, adolescent sexuality leaders and organizations.

The Force Field Analysis framework developed by Kurt Lewin [35] with three dimensions of a goal, driving force towards the attainment of a goal and the restraining forces is employed in this study. The Force Field Analysis states that, behaviour is determined by the totality of an individual's situation. In the field theory, a 'field' is defined as the totality of coexisting facts, which are conceived of as mutually interdependent. It consists of the goal, driving force towards the attainment of a goal and the restraining forces.

The whole psychological field determines the health behaviour manifested. This theory is used to explain the various forces influencing adolescents' ability to abstain from premarital sex and the outcome of not doing so. This model is theoretically justified in the light of factors that shape adolescent decisions on adoption of abstinence. The broad objective of this study was to document the sexual abstinence behaviour of in-school adolescents and identify factors and social context influencing or obstructing their adoption of abstinence as an HIV control measure

## Methods

This descriptive, cross-sectional study was designed to measure knowledge of the causes and prevention of HIV and AIDS; prevalence of abstinence behaviour and factors influencing abstinence behaviour (promoting and obstructing factors) among secondary school students. The study was conducted in Ibadan Southwest, Local Government Area (LGA), and Oyo State, Nigeria. The study area had a total secondary students' enrolment of 882,791 in 2006.

The data was collected using both quantitative and qualitative data collection instruments. Both instruments had been field-tested to ensure their reliability and validity.

For the quantitative survey, a minimum sample size of 383 was obtained using a 5% level of significance, at a 90% power and a 5% tolerable error. The national prevalence of abstinence in males and females was 27% and 47% respectively [11]. This was increased to 420 to adjust for non response. A multistage sampling technique was employed. Fourteen schools were selected proportionally to the school's enrolment size and randomly from a list of 26 coeducational and single-sex public schools in the LGA (5 girls only, 2 boys only and 19 coeducational). Within each selected school, one or two arms of all available classes were randomly selected. This was followed by a systematic random selection of 30 students. The usual age range of students enrolled in Nigerian public secondary school is between 10 and 19 years, however, in some instances; students could be younger or older than this age range. For this study, students who were not within the age range of 10 and 19 years were ineligible and did not participate in the study. Other eligibility criteria considered were consent of the parents and respondents to participate in the study and availability as at the time of the survey.

The selected 420 students later completed a set of semi-structured questionnaire that assessed, among others, their demographic characteristics, sexual activity, self efficacy to refuse sex, social life styles, attitude towards abstinence, knowledge of HIV and AIDS, self esteem, parental control and communication, social approval and peer pressure for premarital sex.

For the qualitative method, eight Focus Group Discussions (FGDs) were conducted. The participants for the FGD were selected randomly in the schools provided they were within the 10 and 19 age group and willing to participate in the discussions. The FGD guide with eleven-questions was designed to elicit the factors associated with practice of primary sexual abstinence, myths as well as approaches to promote abstinence among young people. This tool was used to direct the FGD discussion. The FGDs, which lasted between 50 and 75 minutes, were conducted in English language, though some terms were explained or discussed in the local language. An average of 8-9 respondents participated in each FGD and in all, there were 67 respondents. The FGD groups were stratified based on sex and age (i.e. 10 - 14 females and males and 15 - 19 females and males) and type of school (i.e. single sex or co-educational school). This approach was adopted to ensure homogeneity and facilitate a congenial environment during the discussions. The sessions were moderated by a Facilitator who had been trained to conduct FGDs among young people as well as a note taker to document the verbal responses and non verbal cues during the discussions. With the consent of the discussants, documentation of the FGDs was supplemented with the use of a Tape recorder. At the end of the sessions, light refreshment was provided to the FGD participants. Respondents who participated in the FGD were excluded from participating in the survey due to their prior exposure to information on issues being sought.

Ethical approval for the conduct of the study was obtained from the University of Ibadan (UI) and University College Hospital (UCH) Institutional Review Board, Ibadan, Nigeria. Data gathering process followed standard ethical guidelines. The Local Education Authority and school principals of selected schools were informed and their approval was sought. Parents' written consent was obtained prior to the administration of the questionnaire. The students' consent was obtained orally prior to questionnaire administration and respondents' anonymity was protected by ensuring that no individual identifiers existed in the instruments or in the electronic data set.

The FGD data was transcribed manually, subjected to thematic analysis and summarized by the Moderator. The Force Field Analysis guided the thematic analysis. Factors influencing abstinence were identified and classified as either driving or restraining forces towards the attainment of abstinence in line with the Force Field Analysis. For the quantitative survey, frequency tables were generated for relevant variables. Descriptive statistics such as means and standard deviations were used to summarise continuous variables. Associations between the outcome variable (abstinence) and each explanatory variable were investigated using chi-square test. The significant variables were entered into a logistic regression model to predict the strength of the associations. Odds ratios and 95% CI were computed. Analysis with a probability of .05 or less was considered as significant. The qualitative data was triangulated with the quantitative results

Respondents' knowledge on the modes of transmitting and preventing HIV was assessed using a twenty-question item which had positively and negatively stated questions. For each question, the scale ranged from 0 to 2. The knowledge score was obtained by scoring and summing up all the twenty items, the highest score was 40 while the lowest was 0 [36].

Predictors of sexual abstinence were analysed using logistic regression. Variables analyzed included some selected socio-demographic characteristics (sex, religion, living arrangement, mothers' education), lifestyles (having a boy/girl friend, ever smoked cigarette, ever drank alcohol), and attitude towards abstinence and self esteem.

## Results

All the 14 selected schools in the LGA participated in the study, yielding 420 usable copies of the questionnaires (100% of eligible students). All Parents/guardians consented to their wards participating in the survey.

### Respondents' demographic characteristics

The respondents who participated in the survey were almost evenly distributed by gender (52.1% males and 47.9% females). The mean age was 14.8 years (SD ± 2.2) with most respondents (236 or 56.2%) in the 15-19 year age bracket. These were followed by those aged 10-14 years (43.8%). Majority were Yoruba (78.1%) and Christians (82.9%) with most living with both parents 290 (69.5%) (Table 1). Most respondents' mothers had secondary and post secondary school education (41.9%) and (41.7%) respectively. For the fathers: 36.2% and 57.4% had secondary and post secondary school education respectively.

**Table 1:** Demographic characteristics of respondents

Variable	No (%) n = 420
**Sex**	
Male	219 (52.1)
Female	201 (47.9)

**Age (Years)**	
10-14.	184 (43.8)
15-19	236 (56.2)

**Religion**.	
Christianity	348 (82.9)
Islam	72 (17.1)

**Ethnic Group**	
Yoruba	328 (78.1)
Igbo	55 (13.1)
Edo	13 (3.1)
Hausa	3 (0.7)
Others	21(5.0)

**Person with whom respondents live**	
Both Mother and Father	290(69.5)
Mother	61(14.6)
Father	19(4.6)
Relatives	43(10.3)
Others (Pastor, Teacher)	4(1.0)

**Mothers' level of education**	
No formal education	23(5.5)
Primary	46(11.0)
Secondary	176(41.9)
Above secondary	175(41.6)

**Fathers' level of education**	
No formal education	8(1.9)
Primary	19(4.5)
Secondary	152(36.2)
Above secondary	241(57.4)

### Respondents' knowledge of HIV and AIDS

Most respondents (87.8%) knew HIV could be transmitted through unprotected sexual intercourse; 87.6%, 86.8% and 67.9% stated that it could be transmitted through sharing sharp objects, contaminated blood transfusion and from infected mother to child respectively.

HIV-preventive measures which respondents knew included abstinence (81.0%), using new needles for each injection (77.6%), regular condom use (66.2%) and faithfulness to one partner (60.9%). Majority (81.7%) of male and (81.5%) of female abstinent respondents favoured the promotion of abstinence as an HIV/AIDS prevention strategy compared with 76.0% and 64.0% of those sexually active.

Most respondents had a good knowledge of the modes of HIV transmission and prevention. An overall mean score of 30.6 (SD. 6.8) was obtained on a 40-point scale and the median score was 32 with a range of 40.

### Prevalence of primary abstinence

**Figure 1:**
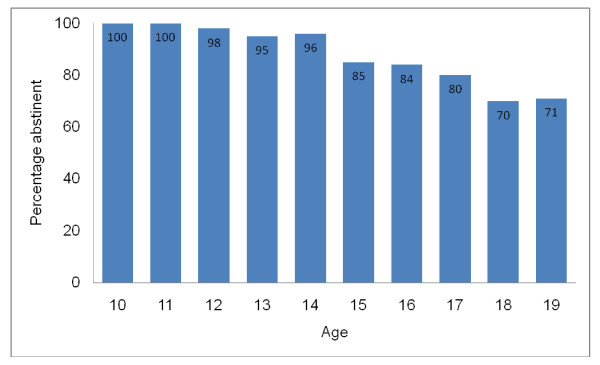
**Percentage of abstinent respondent by age**.

Eighty eight percent of the respondents reported that they had never had sex (primary abstinence), 50 (12%) reported to be non abstinent. The proportion of abstaining male respondents (79%) was smaller than that of abstaining female (98%). More male respondents (11%) than female (1%) were sexually active. The age range of the sexually active respondents was 10 - 19 and percentage of respondent who were abstinent at each age is presented in Figure 1.

### Predictors of sexual abstinence

The factors restraining or driving the adoption of sexual abstinence from the Force Field analysis were analyzed using logistic regression to determine the predictors as well as the odds ratio. The result of the logistic regression showed that the major predictors of abstinence were being a female (OR = 0.2, P = .011, 95%CI = .080 - .657), not having a boyfriend or girl friend (OR = 0.47, P = .000, 95%CI = .022 - .200), not using alcohol (OR = .196, P = .000, 95%CI = .123 to .597) and having favourable attitude (OR = .167, P = .014, 95%CI = .044 to .665) towards abstinence (Table 2 and Table 3).

**Table 2:** Logistic Regression Coefficient of Selected Socio-demographic characteristics, respondent's Lifestyle and positive attitude towards abstinence

Explanatory Variables							95.0% C.I for EXP (B)	
	B	**S.E**.	Wald	df	**Sig**.	OR	Lower	Upper
**Sex**								
Female (RC)								
Male	-1.473	.537	7.516	1	.006**	.229	.080	.657

**Religion**								
Islam (RC)								
Christianity	-0.50	.503	-010	1	.920	.951	.355	2.550

**Does respondent have a boyfriend or a girlfriend**								
No(RC)								
Yes	-2.171	.566	23.064	1	.000**	.066	.022	.200

**Alcohol Use**								
No (RC)								
Yes	-1.307	.403	10.503	1	.001**	.271	.123	.597

**Tobacco or cigarette use**								
No(RC)								
Yes	.057	.979	.003	1	.954	1.059	.156	7.206

**Table 3:** Logistic Regression Coefficient of Selected Socio-demographic characteristics, respondent's Lifestyle and positive attitude towards abstinence

Explanatory Variables							95.0% C.I for EXP (B)	
	B	**S.E**.	Wald	df	**Sig**.	OR	Lower	Upper
**Respondents' closeness to their mother**			2.362	3	.501			
Very close (RC)								
Close	-.624	.467	1.782	1	.182	.536	.214	1.339
Not close	-.318	.876	.132	1	.717	.728	.131	4.049
Can't say	-.848	.784	1.170	1	.279	.428	.092	1.990

**Respondents' closeness to their father**			.033	3	.998			
Very close (RC)								
Close	-.026	.462	.033	1	.954	.974	.394	2.409
Not close	-.117	.663	.031	1	.860	.890	.243	3.260
Can't say	-.086	1.199	.005	1	.943	.918	.087	9.633

**Respondents' attitude towards abstinence**			6.942	2	.031			
Highly favourable (RC)								
Favourable	-1.763	.692	6.498	1	.011**	.172	.044	.665
Unfavourable	-1.241	.735	2.847	1	.092	.289	.068	1.222

**Respondents' self esteem**			1.868	2	.393			
Excellent(RC)								
Good	-.782	.581	1.808	1	.179	.453	.146	1.430
Poor	-.678	.592	1.309	1	.253	.508	.159	1.621

* = P < .05; RC = Reference Category

Perceived self efficacy to refuse sex and perceived non-sexual behaviours of peers were significantly associated with sexual abstinence (p < 0.05) (Table 4).

**Table 4:** Association between perceived self efficacy to refuse sex, peer sexual behaviour and adoption of sexual abstinence

Variable (n = 420)	Abstinence		
	No	Yes	P = value*
	No (%)	No (%)	
**How confident are you to Refuse sex with someone who gives you a lift?**			
Very confident	24 (8.6)	255 (91.4)	
Confident	6 (20)	24 (80)	.014
Not confident	11 (15.5)	60 (84.5)	
Can't say	9 (23.7)	29 (76.3)	

**Attitude towards abstinence**			
Highly favourable	3 (2.7)	109 (97.3)	< 0.001
Favourable	13 (12.4)	92 (87.6)	
Unfavourable	35 (17.9)	160(81.2)	

**Have some of your friends had sexual intercourse?**			
Some of them	23 (19)	98 (81)	
Most of them	9 (27.3)	24 (72.7)	< 0.0001
All of them	8 (42.1)	11(57.9)	
Don't know	10 (4.2)	230 (95.8)	

### Perceived factors influencing the adoption of abstinence

Perceived factors which the abstinent respondents reported during the quantitative survey as influencing their adoption of abstinence include lack of interest or young age (32.5%); fear of HIV/AIDS, STIs, pregnancy and other diseases (23.6%), religious inclination (13.6%) and parental influence and care (6%). (Table 5)

**Table 5:** Perceived factors promoting the adoption of abstinence by non-sexually active respondents

Variable	No (%) n = 370
Religious reasons	50(13.6)
Parental influence/Parental care	22(6)
Fear of HIV/AIDS, STIS, Pregnancy and other diseases	87(23.6)
Want to protect my future/Want to finish my education/Not good for me.	53(14.4)
No opportunity/No boyfriend/girlfriend	6(1.6)
No interest/I am too young.	120(32.5)
Don't want to lose my virginity now/It will pain me if I want to have sex/I want to wait till marriage.	31(8.4)

On the other hand, perceived factors reported by the non abstinent respondents as obstructing their adoption of abstinence include uncontrollable sexual urges (46.7%); peer pressure (17.8%) and financial or material benefits (13.3%) (Table 6). Almost half of the sexually active respondents stated that a factor obstructing their adoption of abstinence was the feeling that sexual intercourse was a normal practice, which young people could not do without.

**Table 6:** Perceived factors obstructing the adoption of abstinence by sexually active respondents

Reasons why respondents found it difficult abstaining	No (%) n = 50
Love	4(8.9)
Uncontrollable sexual urges/it's a normal thing/can't do without it.	21(46.7)
Don't know.	4(8.9)
Material benefits	6(13.3)
Peer pressure/coercion	8(17.8)
I'm old enough./I never plan to abstain	2(4.4)
No response	2

### Factors influencing the adoption of sexual abstinence

The findings from the focus group discussions revealed that in line with the quantitative survey, factors such as age, gender, peer pressure, economic status, religiosity, family background, myths and misconception influenced the adoption of sexual abstinence.

All the groups were in concordance that females and younger adolescents aged 10 to14 years were more likely to abstain from premarital sex.

Major reasons why respondents felt the females abstained more include the fear of pregnancy, sexually transmitted infections, unequal gender norms which label girls who are sexually active as being promiscuous as well as the traditional norms which do not support premarital sex. These views were reflected in the quotes of two-females and one-male discussants in the 15-19 year age group

*"Having sex with a boy is a problem, you don't know the problem with that boy, you only see the face, and you can't see the internal part of his body. So I think the problem is that after you marry, you send it (a sexually transmitted infection) to your husband, so both husband and wife would be carrying the disease*"

"You won't be disgraced, because if you had sex with someone before you married, and later became a millionaire, the first person you had sex with would be saying this girl, I have had sex with her before; she has become a dog to me"

"In Yoruba land (an ethnic group in Nigeria), if a man should marry a girl, and after their marriage, if they went inside and he met the girl (have sexual intercourse), and there is no blood stain on the bed after, which is what signifies that the person is a virgin she and her household will be put to shame"

The groups also stated that males aged 10 to 14 years were more likely to abstain compared to females in that age group due to the fact that older boys would pressurize them to have premarital sex.

Another factor restraining the adoption of abstinence was peer pressure. This opinion was expressed in the quote of a male discussant in the 15-19 year age group. He said

"Show me your friend and I would tell you who you are, it (peer pressure) really affects because if I say I am a saint, but as soon as I see my friends, guys, going around with girls I will feel somehow odd. So, it really affects"

Economic status was also identified as a major influencing factor. Majority of the participants agreed that being economically dependent on a romantic partner hinders adolescents' ability to abstain from premarital sex. Opinions were split on the influence of poverty and the desire to get rich as major influencers. While some participants opined that the poor abstain more due to inability to bear the cost of unwanted pregnancy and of treating sexually transmitted infections, the view point of others was that the poor abstain less because poverty forces them to engage in premarital sex. The rich, according to them, thus exploits the poor.

The opinions expressed by most of the respondents on the influence of economic status are encapsulated in the quotation of a 15-19 year old male. He said *"*I *think the rich and the poor work hand in hand, I have money and I give it to a poor girl who has no money so everything is now "money for hand back for ground" (act of sexual intercourse). So I give her the money and have sex with her. The rich have sex with the poor because the poor needs something"*

All the groups agreed that all religion supports the adoption of abstinence. According to them, Islam and Christianity in particular support abstinence. A 10 - 14 year-old female stated thus

"Being a Christian makes one to abstain and it is in the bible, that though shall not commit fornication - for example in my church, if you are pregnant, my pastor would not join the couple together (conduct the marriage ceremony)"

The groups opined that the media play dual roles. Some Television and radio programmes promote abstinence; on the other hand, some promote premarital sex. The type of programme viewed on the media determines the role it will play in either promoting or obstructing the adoption of abstinence.

Several myths which are widespread among young people were mentioned by the discussants. According to them, this had a great influence on their decision to abstain from premarital sex. The major myths mentioned are as follow:

• If a male does not have sex by age 16, it means he is impotent and may frequently have stomach upset. In addition, he may have a low sperm count and thus, won't be able to acquire the skills needed to have sexual intercourse in the future.

• If a female does not have sex by age 19, her vagina may close up and when she wants to have sex later, it will be painful. She may also be infertile, or have problems having her first child and may have to undergo caesarean operation during childbirth.

### Respondents' suggestion for promoting abstinence

Majority of the students favoured the promotion of abstinence and suggested strategies (interactive multimedia, educative talk and shows, active parent-adolescent communication) in different settings (schools, homes, faith-based institutions). They also emphasized the need for adolescent friendly counsellors and continuous programmes to promote abstinence.

## Discussion

Knowledge of the causes and prevention of HIV among the respondents was high and this is in consonance with several studies [37,38]. This implies that interventions designed to increase knowledge of HIV and related issues have had positive impact.

More respondents in the 10-14 year age bracket abstained from premarital sex compared with those aged 15-19 years. These can be attributed to older adolescents being more vulnerable to extraneous factors such as peer and media influences, lack of parental control and development of secondary sexual characteristics [39-41]. In view of this finding, programmes promoting abstinence must target adolescents aged 15-19 years and also emphasize sex-refusal skills to counter the restraining forces obstructing the adoption of abstinence.

In comparison with the findings of the 2005 National HIV/AIDS and Reproductive Health (NARHS) survey, the respondents in this study had higher abstinence rates. This could be due to their younger age compared with the NARHS survey which had respondents in the 15 and 24 years age group.

The finding that fewer males than females were abstaining from premarital sex seems to mirror the findings from the National HIV/AIDS and Reproductive Health survey that showed a higher prevalence of sexual intercourse among the males [14]. This finding which suggests greater male sexual risk-taking behaviour is hardly surprising given that all sexually active males in the study attributed non-adoption of abstinence to their perception of sexual intercourse being a normal practice which young people could not do without. The widespread perception that men's' sexual needs are beyond their control and demand immediate satisfaction has been reported in other regions [42]. While a higher prevalence of premarital sex among the males has been related to such mistaken beliefs [43], the respondents attributions in this study lend further support to the notion of unequal gender norms that perpetuates a sense of entitlement to sex among young men. This notion reflects the double standards in which virginity is the traditional norm for unmarried girls while young men are expected to be involved in sexual adventure [21].

Given the consistency of this finding with other studies, gender-specific abstinence programs which should address unequal gender norms deserve funding, support and implementation.

The perceived factors obstructing adoption of abstinence, especially romantic love for partners, gifts or monetary rewards and pressure are in consonance with the findings of another study [44] where sexual relationship among pregnant unmarried adolescents attending a maternity facility occurred as a result of material need. These findings further emphasize the need to design intervention programmes that build life skills of young people on poverty eradication, resisting peer pressure, assertiveness, goal setting and negotiation.

By definition, factors are called "protective" if they discourage behaviours that might lead to a negative health outcome. Similarly, factors are labelled "risk factors" if they either encourage or are associated with one or more behaviours that might lead to a negative health factor or discourage behaviours that might prevent them [45]. In this study, predictors of abstinence (being a female, not having a boyfriend or girl friend, not using alcohol and having a good attitude towards abstinence) have been identified.

These findings are consistent with those of other researchers [19,24,46,47] that examined predictors of premarital sex. A thorough understanding of these predictive factors will allow programme managers to develop effective interventions that target those factors known to promote abstinence behaviours, and hence, outcomes.

### Implication for health education

The major factors influencing the adoption of abstinence can be broadly classified into peer influences, unequal gender norms, adolescent social lifestyle, perceived self efficacy to adopt abstinence, attitude towards abstinence and the media. In the light of these findings, comprehensive sexuality education programmes which encourage abstinence and other prevention strategies - mutual fidelity and condom use should be developed and implemented in school settings. This is particularly important in view of the evidences which suggest that abstinence only programmes do not seem to delay sexual debut or reduce the risk of HIV transmission. Other complementary interventions such as peer education and role modelling are needed to address the factors.

Advocacy efforts should target policymakers and gatekeepers to ensure that policies are reviewed to support comprehensive sexuality education in secondary schools. It is also imperative that fund is allocated and released for the implementation of in-school sexuality education programmes.

Training program goal should emphasize the empowerment of young people to develop and acquire personal skills needed to adopt abstinence. The training programmes must not only stress the cognitive aspect of learning but also boost young persons' confidence and upgrade their skills on issues relating to the adoption of abstinence and self efficacy such as assertiveness, refusal skills and goal setting. It should also reinforce the perceived benefits of sexual abstinence.

Diverse behaviour change intervention strategies that would create a favourable attitude towards the practice of the behaviour, promote the values of abstinence and imbibe the necessary skills required to adopt abstinence are essential. These include but are not limited to interpersonal communications, electronic (radio and television phone-in youth programmes) and print media (cartoons, youth-focused magazines, posters, stickers and handbills), peer-education, parent-child communication, and adult-role modelling (through which adults share experiences on how they were able to navigate the period of adolescence with particular emphasis on sexual abstinence). In addition, renowned actors, athletics or musicians can be used as positive role models. All these have the potentials of enhancing young peoples' confidence and imparting the required abstinence skills.

## Conclusions

The study has identified the factors that protect young people from premarital sex and those that put them at risk. Given that sexual behaviour of in-school adolescents are influenced by multiple factors, the researchers hereby recommend an integrated multi-sectoral approach involving all stakeholders in providing comprehensive abstinence sexuality education to young persons.

## Limitation of the study

Self-reported assessments of sexual behaviour are prone to a number of biases that could affect the reliability and validity of a measure ranging from participants literacy level and comprehension of behavioural terminology, to recall biases and self-presentation or confidentiality concerns resulting from stigmatization of the behaviour in question. Our study focused on a sensitive issue considering the fact that stigma is normatively attached to premarital sex among young people in Nigeria.

While this might affect the accuracy and generalizability of the findings, efforts were made to mitigate the impact of this effect by assuring respondents of full confidentiality and by making the questionnaire a guided self-administered process.

Another limitation identified was the study design which employed a cross sectional study in the conduct of the quantitative survey. A key limitation of this study design in assessing predictors of sexual abstinence is the difficulty in differentiating cause and effect from simple association. Cohort studies measure potential causes before the outcome has occurred. This design can demonstrate that these "causes" preceded the outcome, thereby avoiding the debate as to which is the cause and which is an effect. However, the strength of this study which mitigated this factor was the relatively large number of respondents and the good response rate.

The sample was a clustered sample as the primary sampling unit was Schools. However; this was not included as a random effect in the logistic regression analysis and is likely to have resulted in larger estimates of the errors. Therefore, care should be taken in interpreting the errors and p values. This however does not have a major effect on the fairly clear cut results and therefore the interpretation of the study.

## Competing interests

The authors declare that they have no competing interests.

## Authors' contributions

OO conceived and coordinated the conduct of the study, developed draft instruments and wrote the draft manuscript. MMF pre-tested survey instruments, conducted FGDs, wrote drafts of findings. All authors read and approved of the final manuscript.

## Pre-publication history

The pre-publication history for this paper can be accessed here:

http://www.biomedcentral.com/1471-2458/11/304/prepub
